# Supplementary material for: Genome-wide mapping using new AFLP markers to explore intraspecific variation among pathogenic Sporothrix species
Source: PLoS Negl Trop Dis. 2020 Jul 1;14(7):e0008330. doi: 10.1371/journal.pntd.0008330 (PMC7329091; doi:10.1371/journal.pntd.0008330)
Supplement: S1 Table — (PDF) [file pntd.0008330.s001.pdf]

S1 Table. Diversity of AFLP fragments (50-500 bp) based on *in silico* characterization of *Sporothrix* spp. genomes for 256 selective combinations.

**Genome**	**AWTV01**		**AWEQ01**		**AXCR01**		**NTMI01**		**PGUU01**		**LVYW01**		**LVYX01**		**JNEX02**		**AZHD01**	
**Method**	**AFLP**	**ISIF**	**AFLP**	**ISIF**	**AFLP**	**ISIF**	**AFLP**	**ISIF**	**AFLP**	**ISIF**	**AFLP**	**ISIF**	**AFLP**	**ISIF**	**AFLP**	**ISIF**	**AFLP**	**AFLP**
Minimum n. of AFLP markers	0	0	2	1	2	1	3	3	2	3	1	3	2	3	1	2	1	3
Median n. of AFLP markers	11	12	10	12	10	12	11	12	11	12	12	13	12	13	14	15	11	13
Maximum n. of AFLP markers	34	37	36	38	38	39	35	40	35	41	43	46	39	43	56	62	36	42
25% %tile	8	9	7	8	7	8	8	9	8	9	9	10	9	10	10	11	8	9
75% %tile	14	16	14	15	13	15,75	15	17	15	17	15	17	15	17	19	20	14	17
90% %tile	18	21	18	19	18,30	20	19	21,30	19	21	20	22	20,30	23	25	28,30	19	21,30
Mean	11,55	12,90	11,15	12,41	11,04	12,53	12,01	13,44	12,02	13,33	12,61	13,87	12,73	14,01	15,27	17,16	11,73	13,71
Std. Deviation	5,403	6,042	5,462	5,799	5,394	5,830	5,462	6,015	5,626	6,134	5,745	6,362	5,715	6,239	8,398	9,252	5,801	6,142

AFLP: AFLPinsilico software; ISIF: In Silico Fingerprinting software.
