# Supplementary material for: Genome-wide mapping using new AFLP markers to explore intraspecific variation among pathogenic Sporothrix species
Source: PLoS Negl Trop Dis. 2020 Jul 1;14(7):e0008330. doi: 10.1371/journal.pntd.0008330 (PMC7329091; doi:10.1371/journal.pntd.0008330)
Supplement: S2 Table — (PDF) [file pntd.0008330.s002.pdf]

S2 Table. Cluster similarities among all the combinations evaluated.

Cluster	N. of samples	Combination 1		Combination 2		Combination 3		Combination 4		Combination 5		Combination 6	
		CC	Similarity level	CC	Similarity level	CC	Similarity level	CC	Similarity level	CC	Similarity level	CC	Similarity level
S. brasiliensis	9	98	44.14% ± 1.59%	94	50.87% ± 2.15%	93	50.42% ±1.79%	93	45.47% ± 1.71%	93	51.96% ± 2.83%	98	37.53% ± 4.06%
S. schenckii	8	86	38.81% ± 2.58%	91	34.86% ± 0.81%	93	37.12% ± 3.63%	97	45.08% ± 3.33%	95	52.56% ± 1.43%	93	27.44% ± 2.55%
S. globosa	6	89	66.58% ± 1.98%	89	63.60% ± 0.81%	93	74.38% ± 1.82%	95	65.04% ± 1.19%	81	64.42% ± 2.35%	91	42.79% ± 1.61%
S. mexicana	2	-	81.87% ± 0.00%	-	95.68% ± 0.00%	-	92.54% ± 0.00%	-	98.44% ± 0.00%	-	98.20% ± 0.00%	-	89.80% ± 0.00%
S. pallida	1	-	-	-	-	-	-	-	-	-	-	-	-
S. chilensis	1	-	-	-	-	-	-	-	-	-	-	-	-
Overall	27	96	24.70% ± 2.56%	97	22.81% ± 3.29%	97	21.83% ± 4.14%	96	23.37% ± 2.94%	97	24.83% ± 4.19%	96	17.94% ± 3.25%

CC: Cophenetic Correlation Coefficient. The cophenetic correlation is a measure of how faithfully a dendrogram preserves the pairwise distances between the original unmodeled data points.
