# Supplementary material for: Genome-wide mapping using new AFLP markers to explore intraspecific variation among pathogenic Sporothrix species
Source: PLoS Negl Trop Dis. 2020 Jul 1;14(7):e0008330. doi: 10.1371/journal.pntd.0008330 (PMC7329091; doi:10.1371/journal.pntd.0008330)
Supplement: Attachment — Submitted filename: Rebutal Letter PNTD-D-19-02101.docx [file pntd.0008330.s003.docx]

**Federal University of São Paulo**

**Department of Microbiology, Immunology, and Parasitology**

**Cell Biology Division**

São Paulo, April 25, 2020.

Dear Prof. Dr. Todd B. Reynolds

Deputy Editor

PLoS Neglected Tropical Diseases

We are pleased to submit our revised manuscript [PNTD-D-19-02101] entitled **“Genome-wide mapping using new AFLP markers to explore intraspecific variation among pathogenic *Sporothrix* species”** by “Jamile Ambrósio de Carvalho, Ferry Hagen, Matthew C. Fisher, Zoilo Pires de Camargo, and Anderson Messias Rodrigues” for consideration of publication in PLoS Neglected Tropical Diseases. The final version of the revised manuscript has been seen and approved by all authors mentioned above and it has not been submitted or accepted elsewhere.

You will find the point-by-point response to the suggestions requested by the reviewers. We appreciate the kindness and the availability of these reviewers and the modifications along the text will undoubtedly contribute to the improvement of the quality of our manuscript.

We hope you have the interest to publish our manuscript.

Sincerely, on behalf of all authors,

**Prof. Dr. Anderson Messias Rodrigues, Ph.D.**

Federal University of São Paulo – UNIFESP – Brazil

amrodrigues.amr@gmail.com

**Reviewer's Responses to Questions**

Key Review Criteria Required for Acceptance?

As you describe the new analyses required for acceptance, please consider the following:

**Methods**

-Are the objectives of the study clearly articulated with a clear testable hypothesis stated?

-Is the study design appropriate to address the stated objectives?

-Is the population clearly described and appropriate for the hypothesis being tested?

-Is the sample size sufficient to ensure adequate power to address the hypothesis being tested?

-Were correct statistical analysis used to support conclusions?

-Are there concerns about ethical or regulatory requirements being met?

**Reviewer #1:** The Methods meet all criteria.

**Answer:** Thank you very much for your comments.

**Reviewer #2:** An ongoing cat-transmitted Sporotrichosis epidemic in Brazil has stimulated over the past few years research interest in this disease that was largely neglected for the previous 50 years, apart from some reports on individual cases or a few small outbreaks usually linked to contaminated plant material. DNA sequences have revealed that the causal agents of sporotrichosis can be any of several Sporothrix species. There are clear differences in symptoms, mode of transmission, virulence, antibiotic resistance, and geographic distribution of these species. Based on sequences of a limited number of genes, it is also well known that there is intraspecific variation between strains of the same species. With the expansion of the cat-transmitted outbreak in Brazil from Rio de Janeiro to other cities, the risk of the disease being spread to other countries is increasing. In addition, intraspecific genetic variation can be used in population style analytics to indicate origins and routes of movement of fungi. For these reasons accurate and fast diagnosis of the causal agents, down to a specific haplotype, is not only highly informative for researchers, but often crucial in terms of selecting the most appropriate treatment.

The aim of the present study was to develop a DNA barcoding technique that not only could facilitate accurate and fast diagnosis, but also to explore and intraspecies variation. The authors have utilizied the available genome sequences of six Sporothrix species to develop new AFLP markers not only to accurately identify species, but also to assign isolates to intraspecies groups. The study was done with extreme care and thoroughness and I could not find any flaws in experimental design, laboratory techniques, analyses, or the interpretation of data. In addition, the paper was carefully written and thoroughly edited, leaving me as a reviewer with very little to comment on. I wish more manuscripts submitted for review would be so carefully edited.

In the attached pdf I have made some minor suggestions and corrections.

**Answer:** Thank you very much for your comments.

**Reviewer #3:** The objective, experimental design, samples and data analysis were clearly stated and presented.

**Answer:** Thank you very much for your comments.

--------------------

**Results**

-Does the analysis presented match the analysis plan?

-Are the results clearly and completely presented?

-Are the figures (Tables, Images) of sufficient quality for clarity?

**Reviewer #1:** The results are well drafted and clearly illustrated.

**Answer:** Thank you very much for your comments.

**Reviewer #2:** No problems here, see above.

**Answer:** Thank you very much for your comments.

**Reviewer #3:** Results were clearly presented in details.

**Answer:** Thank you very much for your comments.

--------------------

**Conclusions**

-Are the conclusions supported by the data presented?

-Are the limitations of analysis clearly described?

-Do the authors discuss how these data can be helpful to advance our understanding of the topic under study?

-Is public health relevance addressed?

**Reviewer #1:** -Are the conclusions supported by the data presented? YES

**Answer:** Thank you very much for your comments.

-Are the limitations of analysis clearly described? YES

**Answer:** Thank you very much for your comments.

-Do the authors discuss how these data can be helpful to advance our understanding of the topic under study? YES

**Answer:** Thank you very much for your comments.

-Is public health relevance addressed? YES

**Answer:** Thank you very much for your comments.

**Reviewer #2:** No problems here, see above.

**Answer:** Thank you very much for your comments.

**Reviewer #3:** Conclusions are adequate.

**Answer:** Thank you very much for your comments.

--------------------

Editorial and Data Presentation Modifications?

Use this section for editorial suggestions as well as relatively minor modifications of existing data that would enhance clarity. If the only modifications needed are minor and/or editorial, you may wish to recommend “Minor Revision” or “Accept”.

**Reviewer #1:** At line 432 some missing text makes this sentence unclear: "cluster 4, fragments generated varied from 14-44, and S. pallida isolate clustered apart from the S. pallida complex in AFLP combinations #1, #2 and #4."

**Answer:** We agree with this reviewer and we changed it accordingly.

In clade 4, AFLP markers varied from 14-44 fragments, and S. pallida isolate FMR 8803 grouped apart from the other members of the S. pallida complex (i.e. S. chilensis and S. mexicana) in combinations #1, #2 and #4.

The Discussion needs to be trimmed to minimize overlap with the Introduction, e.g., compare lines 94-112 with 556 – 564. The Discussion should not be written as a separate self-contained essay that assumes the reader knows nothing of what's stated in the Introduction and Results.

**Answer:** We agree with this reviewer and we changed it accordingly.

This entire paragraph (lines 556 – 564) was removed from the “Discussion”

“Sporotrichosis has emerged recently with zoonotic transmission through felines caused by *S. brasiliensis* [85], and it is the main agent in South and Southeast Brazil [42, 43, 46, 82]. *Sporothrix brasiliensis* prevalence can be partially explained by successful animal horizontal transmission (cat-to-cat) and zoonotic transmission (cat-to-human) [43]. Most studies performed for *Sporothrix* were unable to assess intraspecific variability in *S. brasiliensis*, and the predominant population structure in *S. brasiliensis* is considered to be clonal [41, 43, 77, 78, 84, 85]. Despite previous studies that considered *S. brasiliensis* as less diverse genetically than *S. schenckii* [41, 43, 84], subtle intraspecific variations among isolates of *S. brasiliensis* were found using T3B PCR fingerprinting to identify species [87, 90]. Our AFLP results confirm that undiscovered variation occurs in *S. brasiliensis*.”

**Reviewer #2:** Just a very few minor editorial corrections needed.

**Answer:** We agree with this reviewer and we changed it accordingly.

**Reviewer #3:** (No Response)

--------------------

Summary and General Comments

Use this section to provide overall comments, discuss strengths/weaknesses of the study, novelty, significance, general execution and scholarship. You may also include additional comments for the author, including concerns about dual publication, research ethics, or publication ethics. If requesting major revision, please articulate the new experiments that are needed.

**Reviewer #1:** Intriguingly novel bioinformatics-based genetic typing technique for epidemiology, and successfully applied.

**Answer:** Thank you very much for your comments.

**Reviewer #2:** See above

**Answer:** Thank you very much for your comments.

**Reviewer #3:** This paper reports development of amplified-fragment-length polymorphisms (AFLP) to assess genetic diversity among Sporothrix species. Whole genome sequences from Sporothrix species were used to generate virtual AFLP fingerprints, which guided the development of 6 primer pair combinations to be tested in wet lab. A total of 27 Sporothrix isolates (S. brasiliensis, S. schenckii, S. globosa, S. mexicana, S. chilensis, and S. pallida) obtained from clinical lesions of patients, animals and environmental sources were analyzed by the newly developed AFLP markers. Three AFLP markers revealed high discriminating power in distinguishing different strains of Sporothrix species that were thought belonging to the same clonal lineages. These genetic markers are suitable to track Sporothrix spp transmission during epizooties and zoonosis, and to understand the ecology and evolution of pathogenic Sporothrix species.

The manuscript reads well. The authors took an in-depth analysis of the newly developed AFLP markers and showed their high discriminatory power to differentiate different strains among Sporothrix species.

I only have minor comments:

In Abstract, added the results from the 27 Sporothrix isolates studied.

**Answer:** Yes, in the abstract we added the results from 27 *Sporothrix* isolates used in this study.

We included (Lines 33-34): The protocol was used to genotype 27 medically relevant *Sporothrix*.

In Figure 7, the topologies of Combinations 1 to 4 are similar, but Combinations 5 and 6 are quite different, any explanation?

**Answer:** We agree with this reviewer. The Minimum Spanning Tree (MST) algorithm allows short-term divergence and micro-evolution in populations to be reconstructed based upon sampled data. In our case, AFLP-derived MSTs were similar in their identification of totally different types of genetic variants. Deviations in distribution patterns can be observed as well. The pattern observed in our analysis is influenced by the number of isolates (n=27) and the diversity of genotypes included. In this scenario, a more robust and reliable tracking will be achieved in the future when hundreds of *Sporothrix* isolates recovered from ongoing epidemics will be included in the MSTs analysis. This analysis will be fundamental to track *S. brasiliensis* emergence in Brazil and other areas of America.

Whereas it is clear that AFLP has many advantages to be used for epidemiology studies, the author may want to discuss its disadvantages, such as the need of DNA sequencing facility (even though not sequenced in this case), and the uncertainty of amplified fragments with similar size but different sequences. The portability of this method will need to be proved by other labs in future studies.

**Answer:** We thank the reviewer for this valuable comment and agree that it would be highly interesting to discuss the AFLP disadvantages.

Disadvantages include the dominance of alleles, and the possible non-homology of comigrating fragments belonging to different loci, leading to suboptimal reproducibility, particularly across different platforms.

--------------------
